# Changes in plasma protein levels as an early indication of a bloodstream infection

**DOI:** 10.1371/journal.pone.0172987

**Published:** 2017-02-24

**Authors:** Pentti Kuusela, Mayank Saraswat, Sakari Joenväärä, Johanna Kaartinen, Asko Järvinen, Risto Renkonen

**Affiliations:** 1 Division of Clinical Microbiology, HUSLAB, University of Helsinki and Helsinki University Hospital, Helsinki, Finland; 2 Department of Bacteriology and Immunology, University of Helsinki, Helsinki, Finland; 3 Transplantation laboratory, Haartmaninkatu 3, University of Helsinki, Helsinki, Finland; 4 HUSLAB, Helsinki University Hospital, Helsinki, Finland; 5 Emergency Medicine and Services, Helsinki University Hospital, Helsinki, Finland; 6 Division of Infectious Diseases, HUH Inflammation Center, University of Helsinki and Helsinki University Hospital, Helsinki, Finland; NIH, UNITED STATES

## Abstract

Blood culture is the primary diagnostic test performed in a suspicion of bloodstream infection to detect the presence of microorganisms and direct the treatment. However, blood culture is slow and time consuming method to detect blood stream infections or separate septic and/or bacteremic patients from others with less serious febrile disease. Plasma proteomics, despite its challenges, remains an important source for early biomarkers for systemic diseases and might show changes before direct evidence from bacteria can be obtained. We have performed a plasma proteomic analysis, simultaneously at the time of blood culture sampling from ten blood culture positive and ten blood culture negative patients, and quantified 172 proteins with two or more unique peptides. Principal components analysis, Orthogonal Projections to Latent Structures Discriminant Analysis (OPLS-DA) and ROC curve analysis were performed to select protein(s) features which can classify the two groups of samples. We propose a number of candidates which qualify as potential biomarkers to select the blood culture positive cases from negative ones. Pathway analysis by two methods revealed complement activation, phagocytosis pathway and alterations in lipid metabolism as enriched pathways which are relevant for the condition. Data are available via ProteomeXchange with identifier PXD005022.

## 1. Introduction

Blood culture is a primary test to reveal the infection etiology in a febrile patient with a suspicion of serious infection and to give important information on the need and quality of antibiotic treatment. However, in most cases, it takes more than 24 hours to reveal a positive finding in blood culture and treatment must be initiated empirically. Depending on the criteria used for taking blood culture, it is positive in less than 10% or maximally in about one third of cases [1, 2]. Biomarkers such as PCT (procalcitonin), CRP (C-reactive protein) and sTREM (soluble triggering receptor expressed on myeloid cells) are shown to be sensitive predictors of bacteremia (Bloodstream infection) [3–5]. However, they may be elevated in less serious infections or systemic inflammatory responses due to other causes than a blood stream infection. The problems to discern patients with a blood stream infection from those with a serious systemic inflammatory response due to other causes like trauma or inflammation led to definition of criteria for systemic inflammatory response syndrome (SIRS) which have guided the treatment and research of serious ill patients especially in intensive care units [6, 7]. Recently, these criteria have been shown to be unable to find all patients with risk of fatal outcome [8]. It has been shown that CRP is a good marker for bacteremia (Bloodstream infection) [9]. Currently, neither clinical diagnostic criteria nor biomarkers can reliably detect patients with a bloodstream infection in a timely fashion that would enable major changes in treatment efforts.

Early appropriate antimicrobial therapy has been shown to be one of the most important denominators of good prognosis in severe blood stream infections [10, 11]. Early recognition of bloodstream infection might help in allocating antimicrobial and supportive treatment to patients with most benefit of it. Genome based techniques have been shown to be more rapid and even more sensitive in verifying bloodstream infection as compared to conventional culture [12–14]. Whereas positive results may be obtained even within 6 hours, these techniques may only detect given species included into the diagnostic setup. Therefore, a biomarker that would reliably and early discern cases with true blood culture positivity might be an additional advantage in clinical practice.

In this paper we present pilot findings of an LC-MS analysis of plasma proteins simultaneous with blood culture sampling to categorize patients with a bloodstream infection positive and negative blood culture. The decision to perform blood culture has been made on clinical basis by the treating physician in a suspicion of a serious infection like endocarditis, bacteremic pneumonia or urosepsis. Twenty patients were recruited to the study, ten of which were clearly blood culture positive for various bacterial species (details in methods) and ten who were blood culture negative. Both group of patients presented at the clinics as febrile. The basic aim is to find a novel marker for the patients in need of immediate antibiotic therapy. We have quantified 172 proteins with two or more unique peptides. Various statistical/mathematical analysis such as ANOVA, Principle component analysis (PCA), Orthogonal Projections to Latent Structures Discriminant Analysis (OPLS-DA) and ROC curve analysis were performed to select proteins features which can classify the two groups of samples (bloodstream infection positive and negative as found by blood culture).

## 2. Materials and methods

### 2.1 Reagents

For plasma preprocessing Pierce SwellGel Blue Albumin Removal Discs, Pierce Centrifuge columns as well as Pierce C18 Spin Columns were purchased from Thermo Scientific (Rockform, IL, USA). The solvents and high-purity HPLC reagents were purchased from Waters (Milford, MA, USA). All other reagents were from Sigma-Aldrich (St. Louis, MO, USA).

### 2.2 Plasma samples

Blood samples were collected to lithium-heparin tubes from adult febrile patients (age 53–91, median age 76) entering the emergency outpatient clinic of Peijas Hospital, Helsinki University Hospital (Vantaa, Finland) and who were, due to clinical signs, subjects for bacterial blood culturing. Blood cultures were taken based on clinical suspicion of a serious infection both from the cases and from the controls on the same grounds. This has now been emphasized in introduction as well as in discussion. An approval for the study was received from the Ethics Committee of Medical Sciences (HUS 169/13/01/2014) and a written informed consent was obtained from all subjects at the time of plasma sample collection. This written informed consent procedure was approved by the ethics committee. Blood samples (3–5 ml) were adjusted to room temperature for 15 min. Subsequently, plasma was separated by centrifugation (1200xg) for 10 minutes at room temperature. Plasma samples were stored at -70°C until tested at the same time. Blood culturing was performed by using BacT/ALERT^®^ FA Plus and BacT/ALERT^®^ FN Plus blood culture bottles (BioMerieux, Durham, NC, USA) and BacT ALERT 3D incubator (BioMerieux). Identification of bacteria in positive blood cultures was done by Vitek MS MALDI-TOF instrument (bioMerieux).

### 2.3 Plasma treatment and protein digestion

The workflow as well as plasma treatment and protein digestion are described in detail by Kontro et al. [15]. Briefly, after thawing of samples albumin was depleted by Pierce SwellGel Blue Albumin removal discs according to the manufacturer’s instructions. Albumin depleted plasma was assayed by BCA assay kit (Pierce, Thermo Scientific, Rockform, IL, USA) for the total protein concentration. Albumin-depleted plasma corresponding to 350 μg of protein was dried in speed vacuum system (Savant, Thermofisher). After dissolving the dried pellets in 6 M urea and 100 mM Tris-HCl pH 7.4, reduction and alkylation were performed by incubating samples first in 10 mM of DTT for 60 minutes at room temperature (RT) followed by incubation in 30 mM iodoacetamide for 60 minutes in the dark at RT. Finally, after consumption of excess of iodoacetamide with 30 mM DTT (60 minutes at RT) samples were diluted 1:10 with ultrapure water (Milli-Q, EMD Millipore corp.) water and digested with trypsin (1:50 w:w trypsin to protein ratio) for 18 hours at +37°C. Samples were cleaned by Pierce C18 columns according to the manufactures protocol. For LC-MS-analysis 30μg of peptides were first dried in SpeedVac, dissolved in 0.1% formic acid containing 12.5 femtomole Hi3 peptide mixture (Waters) per μL and finally stored at -20°C until analyzed.

### 2.4 Liquid Chromatography-Mass Spectrometry (LC-MS) and quantification

#### 2.4.1 UPLC-MS

Four μL samples, equivalent to ~1.4μg total protein, were injected to nano Acquity UPLC (Ultra Performance Liquid Chromatography)—system (Waters Corporation, MA, USA). TRIZAIC nanoTile 85μm x 100 mm HSS-T3u wTRAP was used as separating device prior to mass spectrometer. Samples were loaded, trapped and washed for two minutes with 8.0 μL/min with 1% B. The analytical gradient used is as follows: 0–1 minutes 1% B, at 2 minutes 5% B, at 65 minutes 30% B, at 78 minutes 50% B, at 80 minutes 85% B, at 83 minutes 85% B, at 84 minutes 1% B and at 90 minutes 1% B with 450nL/min. Buffers were made to UPLC-grade chemicals (Sigma-Aldrich, MO, USA); Buffer A: 0.1% formic acid in water and Buffer B: 0.1% formic acid in acetonitrile.

The data was acquired in DIA (data independent acquisition) fashion using HDMSE-mode with Synapt G2-S HDMS (Waters Corporation, MA, USA). HDMSE mode included Ion mobility spectroscopy (IMS). The collected data range was 100–2000 m/z, scan time one second, IMS wave velocity 650 m/s, collision energy was ramped in trap between 20 to 60 V. Calibration was done with Glu1-Fibrinopeptide B MS2 fragments and as a lock mass, Glu1-Fibrinopeptide B precursor ion was used during the runs. The samples were run as triplicates and further analysis was done with, Progenesis QI for Proteomics–software (Nonlinear Dynamics, Newcastle, UK).

#### 2.4.2 Data analysis

The data analysis procedure has been previously described in details[16]. Briefly, the raw files were imported to Progenesis QI for proteomics software (Nonlinear Dynamics, Newcastle, UK) using lock mass correction with 785.8426 m/z, corresponding to doubly charged Glu1-Fibrinopeptide B. Default parameters for peak picking and alignment algorithm were used. The software facilitated the peptide identification with ProteinLynx Global Server and label-free quantification [17]. The peptide identification was done against Uniprot human FASTA sequences (UniprotKB Release 2015_09, 20205 sequence entries) with (CLPB_ECOLI (P63285)), ClpB protein sequence inserted for label-free quantification. Modifications used were as follows: fixed at cysteine (carbamidomethyl) and variable in methionine (oxidation). Trypsin was used as digesting agent and one missed cleavage was allowed. Fully tryptic cleavage specificity was used. Fragment and peptide error tolerances were set to auto and FDR to less than 1%. One or more ion fragments per peptide, three or more fragments per protein and two or more peptides per protein were required for ion matching. The identified proteins are grouped as one according to parsimony principle and also peptides unique to the protein are reported. Parsimony principle states that protein hits are reported as the minimum set that accounts for all observable peptides. Progenesis QI for proteomics does not take a strict parsimonious approach because of over-stringency as has been pointed out before [18]. However, for resolution of conflicts, if two proteins contain some common peptides, protein with fewer peptides is subsumed into the protein with higher number of peptides which are a superset of the subsumed protein’s peptides. All relevant proteins are listed as a group under the lead protein with greatest coverage or the highest score when the coverages of two or more proteins are equal. Quantitation is performed using the lead identity peptide data. More details about this approach can be accessed on the software website (www.nonlinear.com).

The proteins were considered different if they have a fold change 2 or more and an ANOVA p-value 0.05 or less. The ANOVA calculation assumes that the conditions are independent and applies the statistical test that assumes the means of the conditions are equal. The label-free protein quantitation was done with Hi-N method [17]. In every injection the sample contained also 50 fmol of six CLPB_ECOLI (P63285, ClpB protein) peptides (Hi3 E. coli Standard, Waters). Hi3 peptides are used for normalizing the peptide abundancies and relative quantitation was based on all the non-conflicting peptides found. The peptide ranking is done across all the runs. The abundancies of the peptides are averaged to provide a signal to the protein. Workings of the Progenesis softwares have been described in details on the software website (www.nonlinear.com) and also in published literature [19].

Differences between controls and cases were evaluated with ANOVA on a protein-to-protein basis. Principle component analysis was done with Progenesis QI for proteomics. EZinfo 3.0 (Release date Dec 02, 2014, Umetrics, Sweden) is a separate statistical package that can be used with Progenesis QI for proteomics. The data was imported into the EZinfo and supervised OPLS-DA modeling was performed which gave us the variance vs correlation plot (S-Plot). Default parameters were used. Receiver operating characteristic (ROC) curve analysis was also performed on some of the significantly different proteins predicted by S-Plot. Analyse-It program which works with Microsoft Excel was used for making ROC curves with all the default parameters. The mass spectrometry proteomics data have been deposited to the ProteomeXchange Consortium via the PRIDE partner repository with the dataset identifier PXD005022.

### 2.5 Pathway analysis

Integrated Molecular Pathway Level Analysis (IMPaLA) was used for pathway over representation analysis by their web-based service. The method and rationale behind the approach has been published previously [20]. The background set for IMPaLA analysis were all the proteins entities in all the pathways present in the database (~12185 proteins). Ingenuity pathway analysis (Ingenuity Systems, Redwood City, CA) was used for performing core analysis on the proteomic dataset with default parameters of the software. The results (canonical pathways) are presented in the results section as a figure. The background set for IPA analysis was complete IPA knowledgebase.

## 3. Results

### 3.1 Metadata

Ten samples of blood culture positive men and women and ten blood culture negative age and sex matched febrile patients serving as controls were used for the current study. All samples and their technical replicates behaved similarly in terms of chromatographic performance and alignment therefore no sample was excluded from analysis. Based on the blood culture results subsequent to the sampling, patients were divided into two groups. One group was with a positive blood culture report (8 men, 2 women, “Cases”) and another was with negative blood culture report (10 age and sex matched “Controls”). Bacteria identified in blood cultures were *Escherichia coli* (4 samples), *Streptococcus pneumoniae* (2 samples), coagulase-negative S*taphylococcus* (2 samples), *Staphylococcus epidermidis* and *Streptococcus viridans* (1 sample each). The plasma CRP levels of blood culture positive patients ranged from 12 mg/mL to 399 mg/mL. The percent coefficient of variation was found to be 70.65% for the blood cuture positive patients. Among the control patients who had negative blood cultures, four patients had no infection verified and were not treated by antibiotics and their plasma C-reactive protein at the time of blood culture sampling ranged 13–101 mg/L. Two patients had a pyelonephritis (CRP 125 and 225 mg/L). Four patients had a respiratory infection which was supposed to be of viral origin but three of them received oral antibiotics (doxycycline, cephalexin or co-amoxyclavulanate) and their CRP levels ranged 16–101 mg/L.

### 3.2 Proteomic dataset

In all samples combined, 172 proteins were quantified with the criterion of at least two unique peptides per protein (complete data in S1 Table). Maximum fold changes were 8.844 to 1 when the highest mean was set to blood culture positive and from 5.967 to 1.002 when the highest mean was set to control samples. One protein (Apolipoprotein L2) was found in only the case. Top ten most upregulated (Highest mean case) and down regulated proteins (highest mean control) in blood culture positive samples are given in Table 1.

**Table 1:** Top 10 up- and downregulated proteins in plasma of ten patients with a positive blood culture (cases) and ten with a negative blood culture (controls) at the time point of blood culture sampling. The top proteins are according to the fold change in case versus controls. Only the proteins having less than 0.05 ANOVA p values are shown. Peptide count is the total peptides found for corresponding proteins and unique peptides is the number of unique peptides found out of the total peptides. Confidence score, ANOVA p value, maximum fold change, condition of the highest and lowest mean and full name of the protein are given in separate columns.

Accession	Peptide count	Unique peptides	Confidence score	Anova (p)	Max fold change	Highest mean condition	Lowest mean condition	Description
Q9H497	2	2	10.7157	0.001182	8.844932	Case	Control	Torsin-3A
P07339;C9JH19	2	2	11.8647	0.003668	2.631503	Case	Control	Cathepsin D
Q96SN7;C9J2H9;C9J5L2;C9JQR7;C9JUY6	4	3	27.6575	0.000649	2.027496	Case	Control	Protein orai-2
Q9Y4B5;J3QLE1	3	2	20.227	0.010045	1.870043	Case	Control	Microtubule cross-linking factor 1
Q08AH1;E5RFK0	3	3	18.8377	0.019465	1.84317	Case	Control	Acyl-coenzyme A synthetase ACSM1, mitochondrial
A0A087WU16;B2REA4;O75626	4	4	26.6581	7.38E-07	1.77715	Case	Control	PR domain zinc finger protein 1
Q16531	2	2	7.7408	0.034509	1.750108	Case	Control	DNA damage-binding protein 1
P02750	40	37	186.9309	0.00229	1.640414	Case	Control	Leucine-rich alpha-2-glycoprotein
A0A0A0MTD1;A0A0A0MTC4;A0A0A0MTC5;A0A0A0MTC6;E5RJN7;E7EPX0;E7EVJ4;E9PH62;F8VPI7;G5EA18;Q9NUL3	5	4	22.6587	0.013193	1.61796	Case	Control	Double-stranded RNA-binding protein Staufen homolog 2
P04217;M0R009	48	41	261.3448	0.001136	1.596944	Case	Control	Alpha-1B-glycoprotein
O94782;C9JC88;C9JWX4	3	2	16.0733	0.001325	5.967845	Control	Case	Ubiquitin carboxyl-terminal hydrolase 1
G3V1N2;P69905	3	3	31.698	0.000981	5.127688	Control	Case	HCG1745306, isoform CRA_a
A0A075B702	2	2	17.6117	1.07E-05	4.41976	Control	Case	Protein TRAJ41 (Fragment)
E9PLW5;R4GN25	2	2	10.7804	0.003909	4.260173	Control	Case	Putative protein N-methyltransferase FAM86B1
P38935	2	2	9.2636	0.001711	3.612221	Control	Case	DNA-binding protein SMUBP-2
O60309	4	3	18.0095	0.000957	3.304585	Control	Case	Leucine-rich repeat-containing protein 37A3
E9PPB4	2	2	18.3488	0.002286	3.190066	Control	Case	Peroxisomal biogenesis factor 19
Q7Z3I0;A0A075B7G4;Q8IYB9	3	2	22.3658	0.000508	3.14666	Control	Case	Putative uncharacterized protein DKFZp313E1411
H0YFH1	9	2	78.0417	0.005951	2.729399	Control	Case	Alpha-2-macroglobulin (Fragment)
P01617;A0A0A0MTQ6	4	2	37.2069	6.15E-05	2.570449	Control	Case	Ig kappa chain V-II region TEW

Fourteen proteins passed the cutoff of 0.05 value in ANOVA when the highest mean was set to blood culture positive and 39 proteins passed this cutoff when the highest mean was set to controls. ANOVA p values were one of the main criterions to establish the proteins different in the two classes of samples and therefore the proteins having p values more than 0.05 are not shown in Table 1. These proteins are not considered different despite having big fold changes. The number of unique peptides found was from 2 for multiple proteins to 243 for Alpha-2-macroglobulin. The minimum confidence score reported in the current study is 6.13 for Casein kinase I isoform alpha and highest is 1491 for Alpha-2-macroglobulin. The complete data is given in S1 Table.

### 3.3 Principal component analysis (PCA)

**Fig 1:**
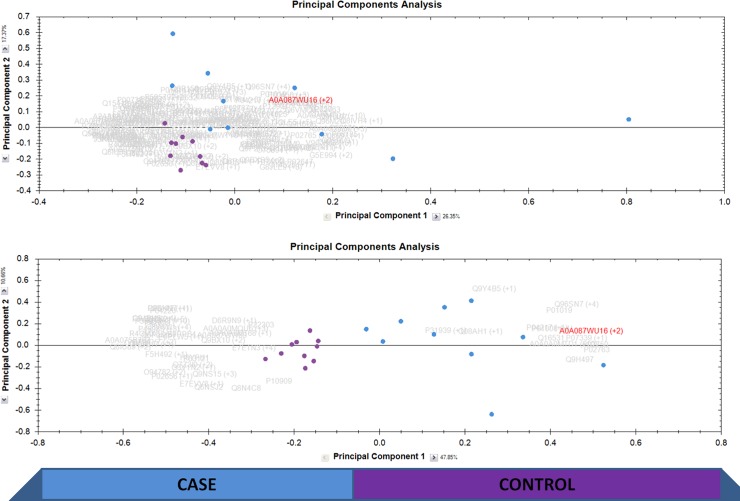
Principal component analysis of the proteomic dataset. Principal component analysis is presented in the figure where purple dots are the controls and blue dots are the cases. The upper panel is when all the proteins with two or more unique peptides were considered for the PCA and lower panel shows the PCA when only the proteins passing the cutoff of ANOVA p value less than 0.05 were considered for PCA. Only one of the triplicate runs are shown in the PCA.

PCA determines the main axes of variations in a dataset, which is helpful in visualization of samples coming from two or more classes. It becomes simpler to visually establish the differences between the classes in a PCA biplot. PCA biplot is presented in Fig 1, when two classes, cases vs controls were used for the PCA. The top panel in PCA represents the condition when all the proteins with two or more unique peptides were considered for PCA and lower panel is when only proteins with fold change of 2 or more between case vs control and also having ANOVA p value less than 0.05 were considered. It can be seen in the top panel that controls are represented in a tight cluster on one side while blood culture positive samples are more spread but separately from the controls. PCA can separate the two classes in Fig 1A. When PCA was performed with proteins already different between the two classes (FC>2 and ANOVA p value<0.05) the separation was even stronger.

### 3.4 Orthogonal Projections to Latent Structures Discriminant Analysis (OPLS-DA)

**Fig 2:**
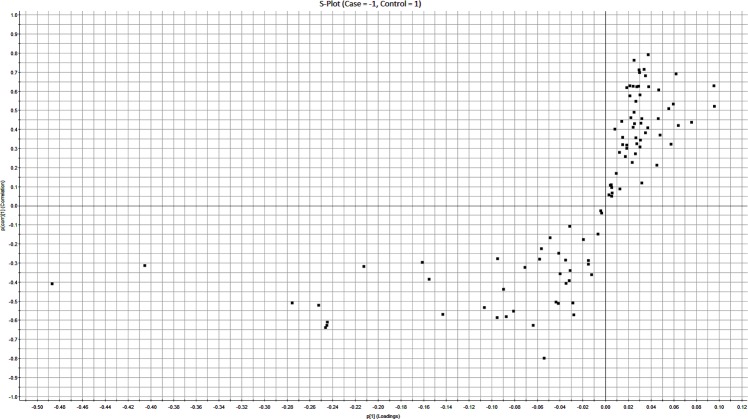
OPLS-DA modeling associated S-Plot. S-plot of the cases with a positive blood culture versus blood culture negative controls: S–Plot obtained from OPLS-DA regression analysis is shown here. Variables having a p(corr) value higher than 0.7 or lower than -0.7 are considered significantly different which can perform class separation between cases and controls. Upper right portions are the proteins downregulated and lower left portion are the proteins upregulated in plasma of patients with a bloodstream infection. Data are log10 transformed and mean centered.

OPLS-DA is a statistical modeling technique which models the data from two classes and presents the differences between the two groups. An S-Plot can be generated which allows for feature selection from the classes which are significantly different among the two groups and can classify them. On the S-Plot, X-axis is the magnitude of change in a particular analyte and Y-axis is the significance of the analyte in comparison of two groups. It can find the predictive and uncorrelated variance in two group comparisons [21]. S-Plot generated for the current study is given in Fig 2. Protein passing the cutoff value of +0.7 or -0.7 for p(corr) values were considered significantly different between the two classes. The proteins passing the cutoff of p(Corr) value higher or lower than 0.7 or -0.7 are presented in Table 2.

**Table 2:** Proteins significantly different in the S-Plot in cases versus controls are shown in the table. Uniprot accession or a group of accessions is given as primary Id. Peptides column represents the total number of peptides found for the said protein and unique peptides are number of unique peptides out of the total peptides for the given protein. Confidence score, ANOVA p value, highest and lowest mean condition, full name of the protein, covariance (p[1]) and correlation (p(corr)[1]) are given in various columns of the table.

Primary accession	All peptides	Unique peptides	Confidence score	ANOVA p-value	Fold change	Highest mean	Lowest mean	Description	p[1]	p(corr)[1]
A0A075B702	2	2	17.6	1.07E-05	4.4	Control	Case	Protein TRAJ41 (Fragment) OS = Homo sapiens GN = TRAJ41 PE = 4 SV = 1	0.03	0.81
E9PLW5;R4GN25	2	2	10.8	3.91E-03	4.3	Control	Case	Putative protein N-methyltransferase FAM86B1 OS = Homo sapiens GN = FAM86B1 PE = 4 SV = 1	0.02	0.80
P01617;A0A0A0MTQ6	4	2	37.2	6.15E-05	2.6	Control	Case	Ig kappa chain V-II region TEW OS = Homo sapiens PE = 1 SV = 1	0.04	0.79
Q8IU89;H0YMG6;H0YN05	4	4	31.0	3.96E-04	1.9	Control	Case	Ceramide synthase 3 OS = Homo sapiens GN = CERS3 PE = 1 SV = 2	0.03	0.79
P38935	2	2	9.3	1.71E-03	3.6	Control	Case	DNA-binding protein SMUBP-2 OS = Homo sapiens GN = IGHMBP2 PE = 1 SV = 3	0.02	0.77
Q7Z3I0;A0A075B7G4;Q8IYB9	3	2	22.4	5.08E-04	3.1	Control	Case	Putative uncharacterized protein DKFZp313E1411 OS = Homo sapiens GN = DKFZp313E1411 PE = 2 SV = 1	0.03	0.76
O94782;C9JC88;C9JWX4	3	2	16.1	1.33E-03	6.0	Control	Case	Ubiquitin carboxyl-terminal hydrolase 1 OS = Homo sapiens GN = USP1 PE = 1 SV = 1	0.02	0.74
Q7RTY7;H0YGY6;H0YH00	6	3	37.5	2.47E-03	2.3	Control	Case	Ovochymase-1 OS = Homo sapiens GN = OVCH1 PE = 2 SV = 2	0.03	0.73
E7EVV8;Q0VG06	2	2	10.9	1.17E-03	1.8	Control	Case	Fanconi anemia-associated protein of 100 kDa OS = Homo sapiens GN = FAAP100 PE = 1 SV = 1	0.03	0.70
P02763	48	38	239.3	6.49E-03	1.6	Case	Control	Alpha-1-acid glycoprotein 1 OS = Homo sapiens GN = ORM1 PE = 1 SV = 1	-0.30	-0.71
P07339;C9JH19	2	2	11.9	3.67E-03	2.6	Case	Control	Cathepsin D OS = Homo sapiens GN = CTSD PE = 1 SV = 1	-0.06	-0.72
P04217;M0R009	48	41	261.3	1.14E-03	1.6	Case	Control	Alpha-1B-glycoprotein OS = Homo sapiens GN = A1BG PE = 1 SV = 4	-0.23	-0.75
P02750	40	37	186.9	2.29E-03	1.6	Case	Control	Leucine-rich alpha-2-glycoprotein OS = Homo sapiens GN = LRG1 PE = 1 SV = 2	-0.23	-0.77
A0A087WU16;B2REA4;O75626	4	4	26.7	7.38E-07	1.8	Case	Control	PR domain zinc finger protein 1 OS = Homo sapiens GN = PRDM1 PE = 1 SV = 1	-0.04	-0.81

### 3.5 Pathway analysis

**Fig 3:**
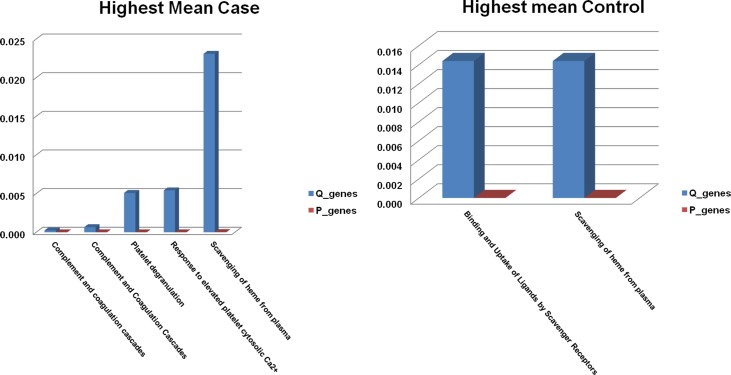
Pathway over-representation analysis by IMPaLA. Pathway enrichment analysis using IMPaLA web based server was performed on two proteins list, one having highest mean in controls (left panel) and other having highest mean in case (blood culture positive). P-values are given in blue bars while Q values are represented by red bars.

We used two different tools for pathway analysis namely Integrated Molecular Pathway Level Analysis (IMPaLA)[20] and Ingenuity pathway analysis (IPA). Pathway overrepresentation analysis was performed using IMPaLA and the results are summarized in Fig 3. When the highest mean was set to controls, only two pathways passed the cutoff (Q_genes of 0.05) which can be seen in the right panel. These pathways were mainly related to scavenger receptors. In the highest mean case condition, several pathways passed this cutoff and top 5 pathways are shown in the Fig 3 left panel. In this category, platelet degranulation and complement and coagulation cascades were the main categories to which most pathways were related. Complement activation will be expected to be overactive in a sepsis like situation and these pathways point towards that phenomenon. All the pathway enrichment results are given in S2 Table.

**Fig 4:**
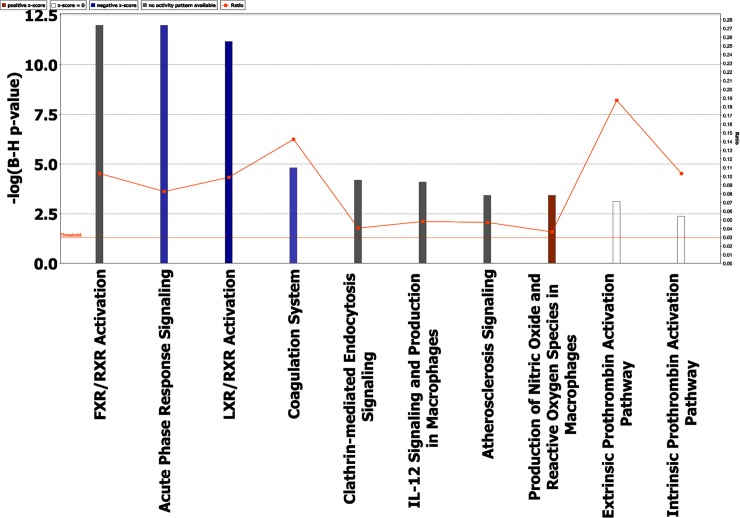
Canonical pathways found by core analysis in IPA. Ingenuity Pathway Analysis “Core analysis” enriched top canonical pathways are shown here. Straight orange vertical line running through the bars is threshold for p value for the particular pathway’s enrichment. Horizontal axis is the–log (p value) and vertical axis represents the given pathways.

Pathway analysis was also performed with IPA tool and several canonical pathways and molecular and cellular functions as well as physiological system development functions as well as networks were enriched in the dataset. Some of these pathways/networks relevant to our study are presented here. Top disease and biofunctions enriched in the dataset are given in S3 Table. Metabolic disease and organismal injury or abnormality were the main category enriched while lipid metabolism, immune response and hematological system development were also enriched (S3 Table). It is to be noted that IMPaLA also enriched the immune response and lipid transport as main categories (Fig 3). Canonical pathways enriched in the current dataset are shown in the Fig 4. Note that only the top pathways are shown due to lack of space in the figure. All the canonical pathways enriched are given in the S4 Table. The canonical pathways in Fig 4 are sorted down to decreasing–log (p value) of enrichment. Note that this p value is corrected for multiple testing (B-H p value). Some of the pathways significant enriched pathways were acute phase response signaling, clathrin-mediated endocytosis, coagulation system and IL-12 signaling.

### 3.6 ROC curve analysis

Proteins selected by S-Plot, as being most significantly different among the two classes of samples (cases vs controls), were further analyzed by ROC curve analysis. Area under the curve (AUC) and optimal cutoff with cost values were calculated which are shown in Table 3. ROC curve is a binary classification technique and it can numerically suggest the better classifiers for two groups among the classifiers analyzed (by AUC values). The program named Analyse-it, was used for calculating these values with default options. These calculations serve as validations of the OPLS-DA model and further suggest which proteins selected by S-Plot also acts as classifiers in ROC curve analysis. One out of the fourteen proteins had the AUC value of 1 and the other eight proteins had the AUC values of 0.90 to 0.99.

**Table 3:** Proteins found to be significantly different in S-Plot were analyzed by ROC curve by Analyse-it program. Uniprot accession, full protein name (description), Area under the curve (AUC), 95% confidence interval (95% CI), standard error (SE), optimum threshold, sensitivity at that threshold and 1-specificity at that threshold and cost for the optimum threshold are shown in the table in various columns.

Primary accession	Description	AUC	95% CI	SE
A0A075B702	Protein TRAJ41 (Fragment)	0.98	0.933 to 1.027	0.02
E9PLW5;R4GN25	Putative protein N-methyltransferase FAM86B1	1.00	1 to 1	0.00
P01617;A0A0A0MTQ6	Ig kappa chain V-II region TEW	0.96	0.886 to 1.034	0.04
Q8IU89;H0YMG6;H0YN05	Ceramide synthase 3	0.94	0.818 to 1.062	0.06
P38935	DNA-binding protein SMUBP-2	0.97	0.911 to 1.029	0.03
Q7Z3I0;A0A075B7G4;Q8IYB9	Putative uncharacterized protein DKFZp313E1411	0.98	0.933 to 1.027	0.02
O94782;C9JC88;C9JWX4	Ubiquitin carboxyl-terminal hydrolase 1	0.97	0.911 to 1.029	0.03
Q7RTY7;H0YGY6;H0YH00	Ovochymase-1	0.87	0.712 to 1.028	0.08
E7EVV8;Q0VG06	Fanconi anemia-associated protein of 100 kDa	0.90	0.748 to 1.052	0.08
P02763	Alpha-1-acid glycoprotein 1	0.83	0.645 to 1.015	0.09
P07339;C9JH19	Cathepsin D	0.89	0.694 to 1.086	0.10
P04217;M0R009	Alpha-1B-glycoprotein	0.85	0.666 to 1.034	0.09
P02750	Leucine-rich alpha-2-glycoprotein	0.86	0.696 to 1.024	0.08
A0A087WU16;B2REA4;O75626	PR domain zinc finger protein 1	0.99	0.962 to 1.018	0.01

## 4. Discussion

Blood culture is currently the gold standard for diagnosing the bloodstream infections. It depends on detecting live microbes in the blood by culturing techniques and many microbes may not be cultivated by the currently used techniques at all. However, even in the best scenarios, the results of the culture are seldom available before 12 hours and in clinical practice much later than that. In most severe cases, after the onset of hypotension in septic shock, every hour’s delay in onset of antibiotic treatment is associated with decreased survival by about 8% [22]. Blood volume used for the culture, time from sampling to incubation, slow-growing microorganisms and ongoing antimicrobial therapy are among some of the factors which influence and reduce the sensitivity of the blood culture [23–29]. There is an increasing need for biomarkers which give an idea of presence of bacterial pathogens in blood stream and type of them even before the blood cultures reports have come-in to aid the clinicians early.

C-reactive protein (CRP) and procalcitonin (PCT) are among the current most promising biomarkers of sepsis. However, CRP for example is increased in several other conditions such as myocardial infarction and trauma [30] and rheumatologic diseases [31]. Non-specific dynamics of CRP don’t support its use as diagnostic biomarker of sepsis. PCT, on the other hand is more specific and several studies demonstrate its elevation in sepsis [32–34]. However, there is controversy regarding its clinical usefulness. A meta-analysis has suggested that PCT cannot differentiate bloodstream infection from other causes of systemic inflammatory response [35]. The lack of appropriate biomarkers to aid in the diagnosis of bloodstream infection calls for more studies to find appropriate biomarkers.

We have performed a pilot study of ten individuals with confirmed blood culture positivity due to various bacteria. Ten age and sex-matched febrile blood culture-negative patients with identical clinical indications for blood culture sampling have served as controls for quantitative plasma proteomics profile. Abundant proteins such as albumin represent 99% of the total plasma proteins and their removal increases the detection of low-abundant proteins which might be clinically important [36]. After the depletion of albumin, we have quantified 172 proteins after filtering to consider only the proteins with two or more unique peptides. These proteins were used for PCA analysis of the samples and it was found that blood culture positive and negative patients separated from each other on the PCA biplot space in two different clusters. The separation was improved even more when only the proteins with FC>2 and ANOVA p-value <0.05 were used for PCA. However, it is to be considered with caution that selecting proteins already different among the two classes for PCA will always lead to better PCA outcomes. Therefore, only the PCA with total proteins should be interpreted in terms of biological meaning in a study.

OPLS-DA modeling and subsequent S-Plot generated the proteins which have the predictive variance for the classes. Only the proteins with the threshold of 0.7 for p(corr) were considered significantly different (Table 2). Among these proteins, was found ceramide synthase 3. One of the family members of this class of protein ceramide sythnase 2 is implicated in sepsis like injury. Cearmide sythnase 2 null mouse die within 10 hr subsequent to injections of high doses of lipopolysaccahrides [37]. Kappa light chains are also previously known to be differentially expressed in different stages of sepsis [38]. However, they have also been implicated in some viral infections such as HIV-1 [39]. They are likely part of the host response during infection and inflammation [40]. Their downregulation in bloodstream infection patients, as found in our dataset (Table 2) represent cases where prognosis might be difficult. Another S-Plot-significant protein found to be downregulated in blood stream infection patients in our dataset was Ubiquitin carboxyl-terminal hydrolase 1 (USP-1). Ubiquitination machinery, particularly ubiquitin ligases, is known to be upregulated in sepsis patients [41]. Therefore it makes sense that in infected patients ubiquitin hydrolase was found to be downregulated in our dataset. Other types of proteases such as serine proteases also play important role in host response against bacteremia [42]. In line with this fact, another S-Plot-significant protein, ovochymase-1 (Table 2) was found to be downregulated in bloodstream infection patients in our dataset. In general, downregulation of host response proteins is a salient feature of bloodstream infection proteins with several examples present in our dataset and that of others. Among proteins, which are upregulated in bloodstream infected patients in our dataset is alpha-1-acid glycoprotein 1 (AGP). This protein is frequently found to be lower in expression in patients which develop septic shock [43]. Patients involved in our study had not reach that stage by the time of sampling therefore, it makes sense that AGP levels were still high. On the other hand, high levels of AGP do not confer any advantages on host and they are deleterious to the survival. It is known that in infected mice (murine model of sepsis), administration of AGP can block the migration of neutrophils to the site of infection, thus increasing mortality [44]. Another upregulated protein in infected patients in our dataset was PR domain zinc finger protein 1 (PRDM1). This protein downregulates the CCL8 which makes the host susceptible to bacterial infections compared to individuals having normal levels of CCL8 [45]. A story emerges from our results containing several examples that host response to infection is extremely important and can dictate the survival of infected individuals. Furthermore, host response, as exemplified by lower or higher expression of its component proteins, is dysregulated in bloodstream infected patients compared to other individuals. These individuals present with fever but remain clearly lower in evolution of infectious disease, not reaching at the level of bloodstream infection at the time of blood sampling. It can be contemplated here that host response is the main differentiating factor between these two groups which is bloodstream infected (group 1) and otherwise febrile patients (group 2). It might be possible to postulate here, that keeping in mind the sensitivity of the blood culture technique, blood based protein biomarkers can be a better indicator of infection status of patients presenting febrile symptoms at the hospital emergency site. At the same time, plasma proteomics combined with statistical analysis can delineate the pathways involved in blood stream infections and make a case for therapeutic target identification in future studies.

Coming to pathways, lipid metabolism and transport was the main molecular and cellular function found by IPA and activation of scavenger receptors was the main overrepresented pathways in the control patients. Complement and coagulation systems pathways were mainly enriched in the case’s proteomic dataset. LXR/RXR activation was one of the pathways enriched in both the IPA and IMPaLA analysis and this pathway is indirectly related to the activation of lipid metabolism and transport. Lipid metabolism was also one of the top molecular and biofunctions in IPA analysis. Altered lipid metabolism has been previously reported in sepsis [46] which is also reflected in our study. Complement and coagulation pathways, on the other hand, are much more obvious and would be expected to be activated upon infection.

The 14 proteins suggested by S-Plot are the high-confidence target biomarkers which can potentially classify patients with a bloodstream infection from blood culture negative control patients. These proteins were further analyzed by ROC curve to validate their predictive power to classify the disease versus healthy control samples. It was noted that, mostly, lower AUC value proteins also had lower p(corr) values. Therefore, these two techniques can prove to be complementary to each other.

In conclusion, we observed clear changes in plasma proteomics among documented blood culture positive patients as compared to blood culture negative patients in this pilot study. These findings seemed not to depend on bacterial species as various different bacteria were found in blood culture positive patients. Among the blood culture negative patients we had a set of real life patients with clear bacterial infections, less clear infectious conditions and clearly patients without any infection and they all differed in their plasma proteomic findings from bacteremic (Bloodstream infected and blood culture positive) patients. These findings encourage further studies to confirm the results in large patient groups and different clinical background. One hope could be that future studies could point out one or more proteins that might be universally differentiated between groups and could be subjected to further development of clinically exploitable and feasible test(s).

## Supporting information

S1 Table Proteins with two or more unique peptides are given here. Accessions (grouped accoprding to parsimony principle), Total peptide count, unique peptides, Confidence score, ANOVA p-value, maximum fold change, conditions of highest and lowest mean and descritpion of protein name are given in various columns. Proteins are sorted according to fold change with highest mean in case followed by highest mean in control.
(XLSX)

S2 Table Pathway over-representation analysis results by IMPaLA are shown in the table. Pathway name, Source, Number of overlapping genes, accession of overlapping genes, number of all pathway genes, P-value and Q values of enrichment are given in the table. This table is for protein list when the highest mean was set to the case. Another list of highest mean control is given in next worksheet in the same file.
(XLSX)

S3 Table Disease and bio-functions enriched in Core analysis of the data by Ingenuity pathway analysis are given in the table along with B-H corrected p values and molecules under each category of bio-functions. (XLSX)

S4 Table All the canonical pathways enriched in the core analysis of the Ingenuity pathway analysis along with -log (B-H p-value), ratio, z-score or activation score and molecules enriched under each canonical pathway are given in the table. (XLSX)
